# Supplementary material for: Evaluation of the Association between Low-Density Lipoprotein (LDL) and All-Cause Mortality in Geriatric Patients with Hip Fractures: A Prospective Cohort Study of 339 Patients
Source: J Pers Med. 2023 Feb 16;13(2):345. doi: 10.3390/jpm13020345 (PMC9967768; doi:10.3390/jpm13020345)
Supplement: Supplementary file 1 [file jpm-13-00345-s001.zip › jpm-2205654-supplementary.pdf]

Table S1. Effects of factors on mortality measured by univariate analysis

	Statistics	HR (95% CI)	*P*-value
Age (year)	80.03±6.46	1.07 (1.04, 1.11)	<0.0001
Sex			
Male	112 (33.04%)	1	
Female	227 (66.96%)	0.74 (0.49, 1.11)	0.1463
Occupation			
Retirement	191 (56.34%)	1	
Farmer	75 (22.12%)	1.06 (0.65, 1.72)	0.8128
Other	73 (21.53%)	0.89 (0.54, 1.49)	0.6671
History of allergy	27 (7.96%)	0.47 (0.17, 1.27)	0.1339
Injury mechanism			
Falling	330 (97.35%)	1	
Accident	5 (1.47%)	0.60 (0.08, 4.32)	0.6134
Other	4 (1.18%)	3.81 (1.20, 12.06)	0.023
Fracture classification			
Intertrochanteric fracture	239 (70.50%)	1	
Femoral neck fracture	92 (27.14%)	0.74 (0.46, 1.19)	0.2121
Subtrochanteric fracture	8 (2.36%)	0.68 (0.17, 2.76)	0.5865
Hypertension	196 (57.82%)	0.76 (0.51, 1.13)	0.1792
Diabetes	70 (20.65%)	1.18 (0.75, 1.87)	0.4791
CHD	166 (48.97%)	1.62 (1.08, 2.42)	0.0187
Arrhythmia	98 (28.91%)	1.95 (1.30, 2.90)	0.0011

Hemorrhagic stroke	9 (2.65%)	0.29 (0.04, 2.07)	0.216
Ischemic stroke	120 (35.40%)	1.10 (0.73, 1.66)	0.6339
Cancer	9 (2.65%)	1.23 (0.39, 3.87)	0.7292
Multiple injuries	20 (5.90%)	0.69 (0.25, 1.88)	0.4674
Dementia	19 (5.60%)	2.60 (1.39, 4.87)	0.0028
COPD	18 (5.31%)	1.00 (0.41, 2.46)	0.9995
Hepatitis	6 (1.77%)	0.53 (0.07, 3.79)	0.5258
Gastritis	7 (2.06%)	0.84 (0.21, 3.41)	0.8062
Time to admission (h)	107.70±359.09	1.00 (1.00, 1.00)	0.7905
Time to operation (d)	4.68±2.31	0.97 (0.88, 1.07)	0.5011
Treatment strategy			
Conservation	23 (6.78%)	1	
ORIF	222 (65.49%)	0.25 (0.15, 0.44)	<0.0001
HA	92 (27.14%)	0.25 (0.13, 0.48)	<0.0001
THA	2 (0.59%)	0.00 (0.00, Inf)	0.9942
Operation time (mins)	99.14±37.30	1.00 (1.00, 1.01)	0.7478
Blood loss (mL)	233.46±144.64	1.00 (1.00, 1.00)	0.2451
Infusion (mL)	1570.70±367.40	1.00 (1.00, 1.00)	0.3681
Transfusion (U)	1.07±1.26	1.04 (0.88, 1.23)	0.6528
Length in hospital (d)	8.46±2.93	1.02 (0.96, 1.09)	0.485
